# Mobile antibiotic resistome in wastewater treatment plants revealed by Nanopore metagenomic sequencing

**DOI:** 10.1186/s40168-019-0663-0

**Published:** 2019-03-21

**Authors:** You Che, Yu Xia, Lei Liu, An-Dong Li, Yu Yang, Tong Zhang

**Affiliations:** Environmental Biotechnology Laboratory, The University of Hong Kong, Pok Fu Lam, Hong Kong

**Keywords:** Nanopore sequencing, Mobile antibiotic resistome, Host-tracking, Genetic context, Conjugative plasmids

## Abstract

**Background:**

Wastewater treatment plants (WWTPs) are recognized as hotspots for horizontal gene transfer (HGT) of antibiotic resistance genes (ARGs). Despite our understanding of the composition and distribution of ARGs in WWTPs, the genetic location, host, and fate of ARGs remain largely unknown.

**Results:**

In this study, we combined Oxford Nanopore and Illumina metagenomics sequencing to comprehensively uncover the resistome context of influent, activated sludge, and effluent of three WWTPs and simultaneously track the hosts of the ARGs. The results showed that most of the ARGs detected in all compartments of the WWTPs were carried by plasmids. Transposons and integrons also showed higher prevalence on plasmids than on the ARG-carrying chromosome. Notably, integrative and conjugative elements (ICEs) carrying five types of ARGs were detected, and they may play an important role in facilitating the transfer of ARGs, particularly for tetracycline and macrolide-lincosamide-streptogramin (MLS). A broad spectrum of ARGs carried by plasmids (29 subtypes) and ICEs (4 subtypes) was persistent across the WWTPs. Host tracking showed a variety of antibiotic-resistant bacteria in the effluent, suggesting the high potential for their dissemination into receiving environments. Importantly, phenotype-genotype analysis confirmed the significant role of conjugative plasmids in facilitating the survival and persistence of multidrug-resistant bacteria in the WWTPs. At last, the consistency in the quantitative results for major ARGs types revealed by Nanopore and Illumina sequencing platforms demonstrated the feasibility of Nanopore sequencing for resistome quantification.

**Conclusion:**

Overall, these findings substantially expand our current knowledge of resistome in WWTPs, and help establish a baseline analysis framework to study ARGs in the environment.

**Electronic supplementary material:**

The online version of this article (10.1186/s40168-019-0663-0) contains supplementary material, which is available to authorized users.

## Background

The emergence and spread of antibiotic resistance genes (ARGs) have raised serious public health concerns. Wastewater treatment plants (WWTPs), as a unique interface between humans and environments, harbor a large microbial genetic diversity, facilitating the exchange of ARGs by horizontal gene transfer (HGT) [1]. Mobile genetic elements (MGEs), including plasmids, integrative and conjugative elements (ICEs; also called conjugative transposons), transposons and integrons, are a means to transfer genetic information between bacterial cells or within the genome of a cell [2]. Importantly, multiple ARGs are often located on MGEs, which makes the transfer of resistance easy to achieve even between bacteria from distant taxonomic lineages [3–5]. Antibiotics and other co-selection factors in sewage form a persistent selection pressure for ARGs and antibiotic-resistant bacteria (ARB) associated with MGEs in the WWTPs [6]. To understand the dynamic dissemination of ARGs and multidrug-resistant ARBs in WWTPs, the ARGs associated with MGEs must be elucidated. Additionally, identifying the hosts of the ARGs is crucial to reveal their fates in the wastewater treatment process.

Previous studies have attempted to reveal the genetic contexts (including MGEs) and hosts of the ARGs with different approaches, including isolation [7, 8], high-throughput sequencing [9, 10], and epicPCR (emulsion, paired isolation, and concatenation PCR) [11]. Pure culture isolation, combined with whole-genome sequencing, has been and remains an important method to determine the phenotypic and genotypic correlations for ARBs and identify the MGEs with which they are associated [12, 13]. However, only a limited fraction of bacteria in WWTPs can be cultured and isolated, which seriously limits the application of pure culture isolation to explore the environmental resistome. High-throughput sequencing has greatly increased our knowledge of the diversity and abundance of ARGs in WWTPs [14, 15]. However, the information about the ARG-carrying species and the genetic contexts remains poorly understood, because of the short read length generated by Illumina sequencing. Although assembly of short reads might provide such information, the frequent repetitive sequences flanking ARGs carried on MGEs usually hamper the effective assembly of genetic contexts of ARGs [16]. EpicPCR was developed to link functional genes and phylogenetic markers, such as 16S rRNA in single cells, and has been used to identify the hosts of ARGs [11]. However, this PCR-based method requires the sequence information of the target genes of interest, and only one functional target gene can be sorted each time, although the phylogenetic marker gene could be a universal one [17]. Above all, a robust method is urgently required to resolve both the genetic environments and the hosts of the ARGs in a high-throughput format.

Third-generation sequencing technologies, including Pacific Biosciences (PacBio) and Oxford Nanopore sequencing, generate long reads (up to 2.27 Mb, as reported) that can span most repetitive sequences and provide an opportunity to link the ARGs and their flanking regions, and thus, the knowledge and technology gap mentioned above can be bridged. Compared with the PacBio platform, Oxford MinION has the advantages of producing raw data in real time and it is a more easily accessible and efficient tool for genome assembly and complex structural detection [18–20].

In this study, we report on the first workflow based on Nanopore and Illumina sequencing to rapidly profile the genetic location and track the hosts of ARGs, particularly for potential ARG-carrying pathogens along the wastewater treatment process. Additionally, the correlation between phenotype and genotype of multidrug-resistant bacteria in both influent and effluent of three WWTPs was determined based on the combination of cultivation and Nanopore sequencing.

## Methods

### Sample collection and pretreatment

Nine samples (i.e., influent, activated sludge, and effluent) were collected from three full-scale wastewater treatment plants (WWTPs) in Hong Kong: Shatin STP (22.407° N, 114.214° E), Shek Wu Hui STP (22.510° N, 114.119° E), and Stanley STP (22.219° N, 114.210° E). The influent and activated sludge samples were fixed on site using an equal volume of 100% ethanol. Additionally, 50 mL of influent without fixation was collected from each of the WWTPs for further multidrug-resistant bacteria isolation. All the samples were transported to the lab for immediate processing (within 2 h). For each influent sample, pellets were collected after centrifuging 50 mL of the sample at 5000 rpm for 15 min at room temperature. For each effluent sample, solids were collected after filtering 1 L of the sample through a 0.45 μm cellulose nitrate membrane. All the processed samples were stored at − 20 °C before DNA extraction.

### Cultivation of multidrug-resistant bacteria

One hundred microliters of each influent without ethanol fixation was plated onto Lysogeny broth (LB) agar plate supplemented with 100 mg/L ampicillin, 50 mg/L kanamycin, 20 mg/L tetracycline, and 25 mg/L chloramphenicol. For the effluent samples, the bacteria pellets on the membranes were resuspended in 1 mL of LB medium, and 100 μL of the resuspension was plated onto the same kind of LB agar plate as described above. After incubating overnight (12 h) at 37 °C, the mixed multidrug-resistant culture used for DNA extraction and downstream analysis for each sample was collected by washing the colonies/isolates grown on the incubated LB plate (approximately 500 colonies/isolates) three times using LB.

### DNA extraction, size selection, and purification

DNA extraction for the influent, activated sludge, and effluent samples was conducted using FastDNA® Spin Kit for Soil (MP Biomedicals, USA) following the manufacturer’s instructions. Total genomic DNA of the mixed isolates from each plate was extracted using a DNeasy PowerSoil Kit (Qiagen, Germany). Equal amounts of DNA from each of the three influent cultures from the three WWTPs were mixed; the same procedure was also conducted for the effluent cultures. After electrophoresis, DNA fragments larger than 8 kb were manually excised from the agarose gel and recovered using the Monarch® DNA Gel Extraction Kit (NEB Inc., USA). The recovered DNA was purified using AMPure XP beads (Beckman Coulter). DNA concentrations and purity were determined using microspectrophotometry (NanoDrop ND-1000; Wilmington, DE). DNA samples of sufficient purity (OD 260/280 of ~ 1.8 and OD 260/230 of 2.0–2.2) were used for library preparation.

### MinION library preparation and sequencing

The sequencing library preparation was performed using SQK-LSK108 1D ligation genomic DNA kit following the procedures described below. For each DNA sample, 1.5–2.0 μg of DNA recovered from agarose gel purification was used.

1. End-repair and dA-tailing were performed using the NEBNext Ultra II End-Repair/dA-tailing Module. In detail, 7 μL of Ultra II End-Prep buffer, 3 μL of Ultra II End-Prep enzyme mix, 5 μL of NFW, and ~ 1.2 μg of DNA were mixed and incubated at 20 °C for 20 min at first followed by another incubation at 65 °C for 15 min.
2. Sixty microliters of AMPure XP beads was used for purification.
3. Ligation was performed by adding 20 μL of Adaptor Mix and 50 μL of Blunt/TA Ligation Master Mix to the 30 μL of dA-tailed DNA and then incubation was performed at room temperature for 15 min.
4. Another purification for the removal of remaining adapters from the adapter-ligated DNA was conducted using 40 μL of AMPure XP beads and ABB buffer supplied in the kit. The purified-ligated DNA was resuspended using 25 μL of ELB, and then the concentration was measured by Qubit to ensure ≥ 500 ng of DNA was retained.

Finally, MinION sequencing was performed using R9.4 flow cells (FLO-MIN 106). A total of eleven independent MinION runs were conducted for all the samples.

### MinION data analysis

Raw reads generated by MinKNOW were base-called using Albacore (v2.1.10) to return fastq files. Passed reads were trimmed for adapters using PoreChop (0.2.3, https://github.com/rrwick/Porechop), and the parameter “--discard_middle” was used to remove the reads with internal adapters. Statistical analysis of the MinION sequencing data was generated and visualized using NanoPack [21]. The base-called data sets were deposited into the NCBI SRA database with the following accession numbers: SAMN09603371-SAMN09603381.

To identify the antibiotic resistance genes, 1D reads after adaptor removal were aligned to the nucleotide sequences of the SARG database [22] using the LAST tool (version 926), which was recommended for high error rate reads with settings as “-a 1 -b 1 -q 2” [23]; next, the aligned result was filtered based on a strict cutoff of alignment length and similarity. Only alignment length cover > 95% of ARG length with similarity > 80% was used for the analysis. When overlapped ARG-containing regions (> 80%) were detected, only the best ARG hit was kept for this region. Then, PlasFlow [24] was used to identify the ARGs carried by plasmids from all the detected ARG reads. Taxonomic identification of the ARG-carrying reads was conducted using Centrifuge (v1.0.3) [25] with the NCBI nonredundant nucleotide sequence database; the classification results were visualized with Pavian (https://github.com/fbreitwieser/pavian). Identification of transposons and integrons located on the ARG-carrying reads was performed using the LAST tool (version 926) to align the sequences to the concatenated protein database of the NCBI Reference Sequence Database (RefSeq), and only the alignments showing 60% [26] amino acid identity over more than 60% of the marker protein (transposase or integrase) length were kept. The ICE-carrying ARGs were determined based on similarity alignment (> 80%) against the ICEs database downloaded from ICEberg [27] and pre-filtered by the criterion of > 50% alignment length over the ARG-carrying reads and then double-confirmed by cross-validation using a blast search against the NCBI non-redundant nucleotide sequence database and manual inspection.

### Illumina sequencing

The extracted DNA (~ 5 μg for each sample) was sent out for high-throughput metagenomics sequencing on the Illumina Hiseq4000 platform using the PE150 strategy at the Novogene Corporation (Beijing, China). On average, 14.5 Gb reads were generated for each sample, and all the sequenced datasets were deposited into the NCBI SRA database (PRJNA505617).

### Illumina data analysis

For each metagenome data, raw reads were filtered to remove those reads containing low-quality (Qscore ≤ 5) base which is over 50% of the total base, adapters, and ambiguous bases (*N* > 10%) [28]. Clean data generated from each sample was de novo assembled using CLC’s Genomic Workbench (version 6.04, QIAGEN Bioinformatics, Denmark) with the default parameters [29], yielding a total of 4,062,135 contigs. Open reading frames (ORFs) were predicted for each assembled contig set using Prodigal (v2.6.3). Then the ARGs-like ORFs were determined using BLASTN against SARG nucleotide database mentioned above at *E* value ≤ 10^−7^ [30] with a minimum similarity of 80% over 95% query coverage. PlasFlow [24] was used to predict plasmid sequences for all ARGs-carrying contigs. To compare the taxonomic affiliation between Illumina and Nanopore sequencing platforms for the mixed influent multidrug-resistant cultures, Centrifuge [25] was also used for the classification of Illumina metagenomic sequences. In addition, this metagenomic data was used for community profiling with Ribotagger [31] using reads annotated as 16S rRNA V4 and V6 regions. Additionally, EMIRGE [32] was applied to reconstruct near-full-length 16S rRNA sequences with 40 iterations. The resulting 16S rRNA sequences were phylogenetically annotated via NCBI-nt database (online nucleotide BLAST) and the relative abundance of the reconstructed genes was estimated by utilizing the probabilistic accounting of reads in EMIRGE.

### Comparison of resistome profiles based on Nanopore and Illumina sequencing

To compare the results generated from Illumina and Nanopore sequencing, ARG number per million base pairs was used for the quantification. Briefly, after the ARGs-like reads were extracted, the ARGs abundance was calculated and normalized by the length of the reference genes in SARGs database and the total sequencing depth. Then the data was analyzed by Pearson correlation coefficient, as well as linear regression analysis.

## Results and discussion

### MinION and Illumina sequencing read statistics

Nanopore sequencing on average generated 3.4 Gb of base-called data with an N50 range from 5872 to 10,674 bp for the nine metagenomics libraries, and the longest length of a single read was 73,530 bp. Compared with these direct sequencings of environmental samples (Additional file 1: Table S1 and Figure S1), the two sequencing libraries constructed for the multidrug-resistant bacteria cultured from influent and effluent yielded much longer reads as indicated by N50 (16,578 bp) with relatively high throughput (avg. 3.9 Gb per flow cell), but even longer reads will likely be achievable by choosing different strategies in DNA extraction and library preparation methods. For example, DNA fragment length will be limited to ~ 60 kb when using spin column kits, whereas larger DNA size can be obtained when using kits based on gravity flow columns (100–200 kb) or traditional phenol-chloroform method (> 150 kb) [33]. Notably, the Nanopore reads length (avg. 5.3 kb; N50 8.1 kb) was much longer than those contigs (avg. 1.4 kb; N50 1.7 kb) assembled using Illumina short reads even the sequencing depth of Illumina sequencing was as deep as 14.5 Gb (Additional file 1: Table S1). These longer Nanopore reads facilitated profiling genetic location of ARGs and tracking their hosts in WWTP microbiomes in this study.

### Plasmids and ICEs carrying ARGs dominate the resistome in WWTPs

As shown in Additional file 1: Table S2, 1791 ARGs-carrying Nanopore reads with an average length of 8.5 kb were identified, much more than the 316 ARGs-carrying contigs (avg. 2.9 kb) assembled from Illumina short reads, indicating the difficulty to assess genetic context of ARGs by assembling Illumina short reads into long contigs.

**Fig. 1:**
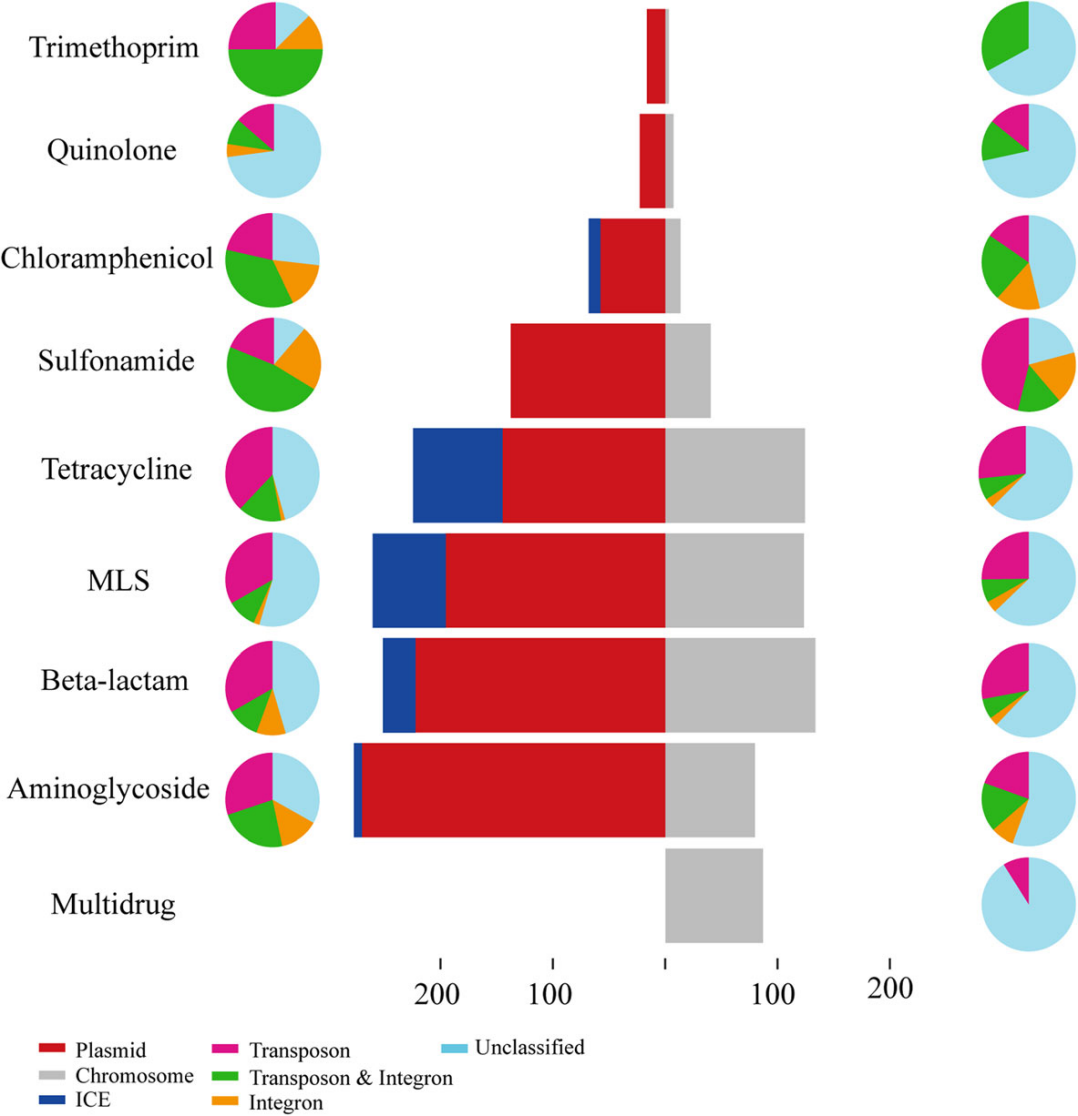
Genetic location of antibiotic resistance genes (ARGs) predicted from the nine Nanopore metagenomic datasets of environmental samples. The resistome was presented by a total of 1791 ARGs-carrying long reads. Bar chart: distribution and abundance of ARGs carried by plasmids, integrative and conjugative elements (ICEs) and chromosome; pie chart: distribution pattern of transposons and integrons located on plasmids (left) and chromosome carrying reads (right)

The resistome of influent, activated sludge, and effluent of the WWTPs presented by the total 1791 ARG-carrying long reads and 316 contigs of the nine environmental metagenomic samples were categorized into two major groups based on their HGT potentials, respectively: (1) the intercellularly mobile group, i.e., ARGs carried by plasmids and ICEs that could be transferred between bacterial cells through transformation or conjugation, and (2) the chromosomal group, i.e., ARGs located on chromosomes that cannot transfer themselves unless integrated into the members of the first group. As shown in Table 1, ARGs in the mobile group on average accounted for 55% of the total number of ARGs revealed by Nanopore sequencing, whereas those on the chromosome accounted for 29%, in addition to 16% that could not be clearly assigned to either of these two groups. Not surprisingly, nearly all types of ARGs in the SARG database were detected in the mobile group, demonstrating the wide distribution of these ARGs on plasmids and ICEs (Fig. 1). Although, several ARGs types in some samples were only detected by Illumina reads (Additional file 1: Table S3), the relative abundance of these ARGs not detected by Nanopore sequencing were very low (accounted for only 4% of total resistome revealed by Illumina sequencing on average). This result indicated that even though the sequencing throughput was relatively low for Nanopore sequencing at the current stage, major ARG types could be sufficiently covered based on the current depth. Note that, except for multidrug ARGs, all other detected ARGs, including aminoglycoside, macrolide-lincosamide-streptogramin (MLS), beta-lactam, tetracycline, sulfonamide, chloramphenicol, quinolone, and trimethoprim, were carried mostly by plasmids, indicating that plasmids were a substantial part in the resistome of the WWTPs (Fig. 1). These findings were in consistence with the results of Illumina assemblies (Additional file 1: Table S4). The quantitative result obtained in this study using Nanopore sequencing further expand the previous descriptive findings that the microbial community of WWTPs can contain a significant collection of plasmids encoding resistance to nearly all clinically relevant antibiotics [34–37].

**Table 1:** Relative abundance of antibiotic resistance genes (ARGs) carried by plasmids, integrative and conjugative elements (ICEs), and chromosome revealed by Nanopore sequencing in different compartments (including influent, activated sludge, and effluent) of three WWTPs

Sample	Plasmids and ICE carrying resistance percentage (%)	Chromosome carrying resistance percentage (%)	Unclassified (%)
STIN	55%	32%	13%
STAS	45%	36%	19%
STEFF	62%	21%	17%
SWHIN	54%	31%	15%
SWHAS	53%	30%	17%
SWHEFF	66%	25%	9%
STLIN	57%	29%	14%
STLAS	41%	33%	26%
STLEFF	65%	22%	13%
Average	55%	29%	16%

*STIN* Shatin STP influent, *STAS* Shatin STP-activated sludge, *STEFF* Shatin STP effluent, *SWHIN* Shek Wu Hui STP influent, *SWHAS* Shek Wu Hui STP activated sludge, *SWHEFF* Shek Wu Hui STP effluent, *STLIN* Stanley STP influent, *STLAS* Stanley STP activated sludge, *STLEFF* Stanley STP effluent

In addition to the ARGs carried by plasmids, 13 ARG subtypes belonging to 5 ARG types were carried by ICEs (Fig. 1), including aminoglycoside (e.g., *aadE*), beta-lactam (e.g., *CfxA*, *CfxA2*, and *CfxA3*), chloramphenicol (e.g., *catQ*), MLS (e.g., *ermB*, *mefA/E*, *ermF*, *ermG*, and *ermC*), and tetracycline (e.g., *tetQ*, *tetM*, and *tetO*). Much more striking was that tetracycline and MLS located on ICEs had a relatively high abundance compared with that of other ARG types, accounting for 22% and 17% of the corresponding ARG type, respectively. Hence, ICEs may play an important role in the spread of ARGs, particularly for those belonging to tetracycline and MLS in WWTPs. The ARGs carried by ICEs revealed in this study were reported pivotal in driving the emergence of multidrug-resistance in diverse gram-positive and gram-negative pathogens [38–40].

ARGs carried by transposons (excluding conjugative transposons) and integrons are incapable of moving intercellularly, but they can “hitch a ride” on the intercellular mobile elements and therefore can further increase the possibility of HGT. As shown by the distribution patterns on both plasmids and chromosomes (Fig. 1), generally, the transposons and integrons were more frequently detected associated with ARGs on plasmids rather than on the chromosome. Six ARG types (trimethoprim, chloramphenicol, sulfonamide, tetracycline, beta-lactam, and aminoglycoside) carried by plasmids were closely related to (> 50% reads in each type) transposons and intergrons, whereas two types (chloramphenicol and sulfonamide) were detected on the chromosome. Thus, the combination of these elements creates an ideal environment on plasmids that enables ARGs to be quickly recruited and accumulated through transposition and recombination [41, 42]. Collectively, these results demonstrated that the ARGs carried by plasmids and ICEs dominated the resistome in the WWTPs and that the transposable elements and integrons might further increase the mobility of plasmid-mediated ARGs.

As to the occurrence and dynamics of the ARGs across the treatment process, notably, the effluent had an even higher proportion of plasmids and ICEs carrying ARGs (ranging from 62 to 66%) than that of the influent (ranging from 54 to 57%, *P* < 0.01 by *t* test) and activated sludge (ranging from 41 to 53%, *P* < 0.01 by *t* test) (Table 1). This result was in consistence with the recent findings that the relative abundance of MGE-associated ARGs increased in the effluent compared with influent [43]. The increase in proportion observed in the effluent might be an indication of the threat posed by the effluent of WWTPs on the dissemination of MGE-associated ARGs in the receiving water [44, 45].

### Prevalence and persistence of ARGs revealed by MGEs and host-tracking

**Fig. 2:**
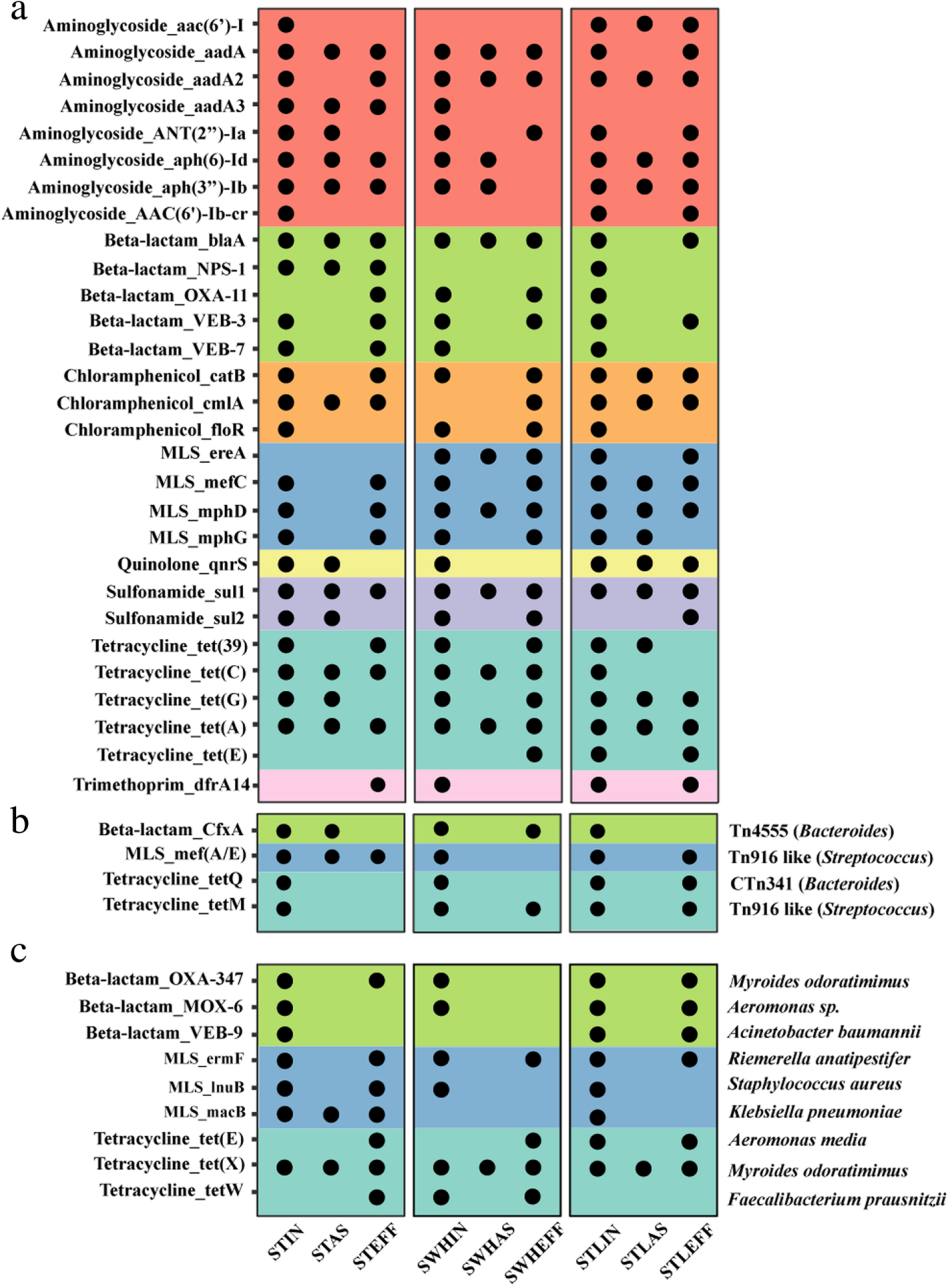
Prevalence and persistence of resistome across wastewater treatment compartments revealed by mobile genetic elements (MGEs) and host tracking, the black dots indicate presence of a specific ARG subtype in different samples, only ARG subtypes detected in both influent and effluent in at least one of the three WWTPs were shown. **a** ARG subtypes carried by plasmids. **b** ARG subtypes carried by ICEs. **c** ARG subtypes located on the chromosome. The same color represents same ARG type

Because of the remarkable resistance mobility observed in this study, we then tracked the ARGs associated with MGEs and hosts of ARGs localized on the chromosome through the treatment process (Fig. 2). A total of 29 ARG subtypes belonging to 8 types carried by plasmids were identified in both influent and effluent in at least 1 of the 3 WWTPs (Fig. 2a). Of the 29 ARG subtypes, 23 were shared by all the WWTPs, and 10 (e.g., *aadA*, *aadA2*, *blaA*, *VEB-3*, *catB*, *cmlA*, *mefC*, *mphD*, *sul1*, and *tetA*) were detected in all 3 effluents. Particularly, the *tetA* (tetracycline-resistant) gene and *sul1* (sulfonamide-resistant) gene carried by plasmids had a persistent prevalence in all the compartments of the three WWTPs. These results are consistent with previous works that many plasmids isolated from WWTPs encode most of the persistent ARGs detected in this study [34, 46]. However, only based on metagenomic sequencing, it is currently impossible to determine the plasmids hosts due to their ability to move across different species. In future studies, Nanopore sequencing, combined with other technologies, such as high-throughput chromosome conformation capture (Hi-C) could help to bridge the knowledge gap.

ARGs carried by ICEs were also tracked across the treatment process. Four ARGs, including *cfxA* (extended-spectrum-beta-lactam-resistant), *mefA/E* (macrolides-resistant), *tetQ* (tetracycline-resistant), and *tetM* (tetracycline-resistant), were detected in all three WWTPs (Fig. 2b). Both *cfxA* and *tetQ* genes were carried by ICEs from *Bacteroides*. It has been reported that *tetQ* in *Bacteroides* has increased dramatically from approximately 30% to more than 80% because of HGT [47], and the involvement of Tn4555 in spreading the *cfxA* gene in *Bacteroides* species has also been confirmed [48]. As the most predominant anaerobes in the human colon (~ 25–30%), *Bacteroides* may serve as reservoirs of ARGs when being released into the WWTPs. Additionally, Tn916-like ICEs from *Streptococcus* carrying *mef*(*A*/*E*) and *tetM* genes were detected. This ICE family that harbors a variety of ARGs is found in an extremely diverse range of bacteria and has the potential to mobilize non-self-transmissible elements [49–51]. Therefore, the ICEs are widespread in WWTPs, making them important vectors in the dissemination of various ARGs between human pathogens and environmental bacteria.

Similarly, the hosts and distribution patterns of ARGs located on the chromosome were also determined (Fig. 2c). The results showed that hosts of all identical ARG subtypes (e.g., *OXA*-*347*, *MOX*-*6*, *VEB*-*9*, *ermF*, *lunB*, *macB*, *tetE*, *tetX*, and *tetW*) across the WWTPs were attributed to seven genera and showed high stability (existing in both influent and effluent) in all the three WWTPs. For example, *ermF* (macrolides-resistant) and *tetX* (tetracycline-resistant) carried by *Riemerella anatipestifer* and *Myroides odoratimimus*, respectively, were detected in all influent and effluent samples. Overall, our results demonstrated that a high diversity of ARGs persisted through the treatment processes in WWTPs, and their association with plasmids and ICEs might have a large contribution to the spread of ARGs.

### Rapid deciphering of potential antimicrobial-resistant pathogens in WWTPs

**Fig. 3:**
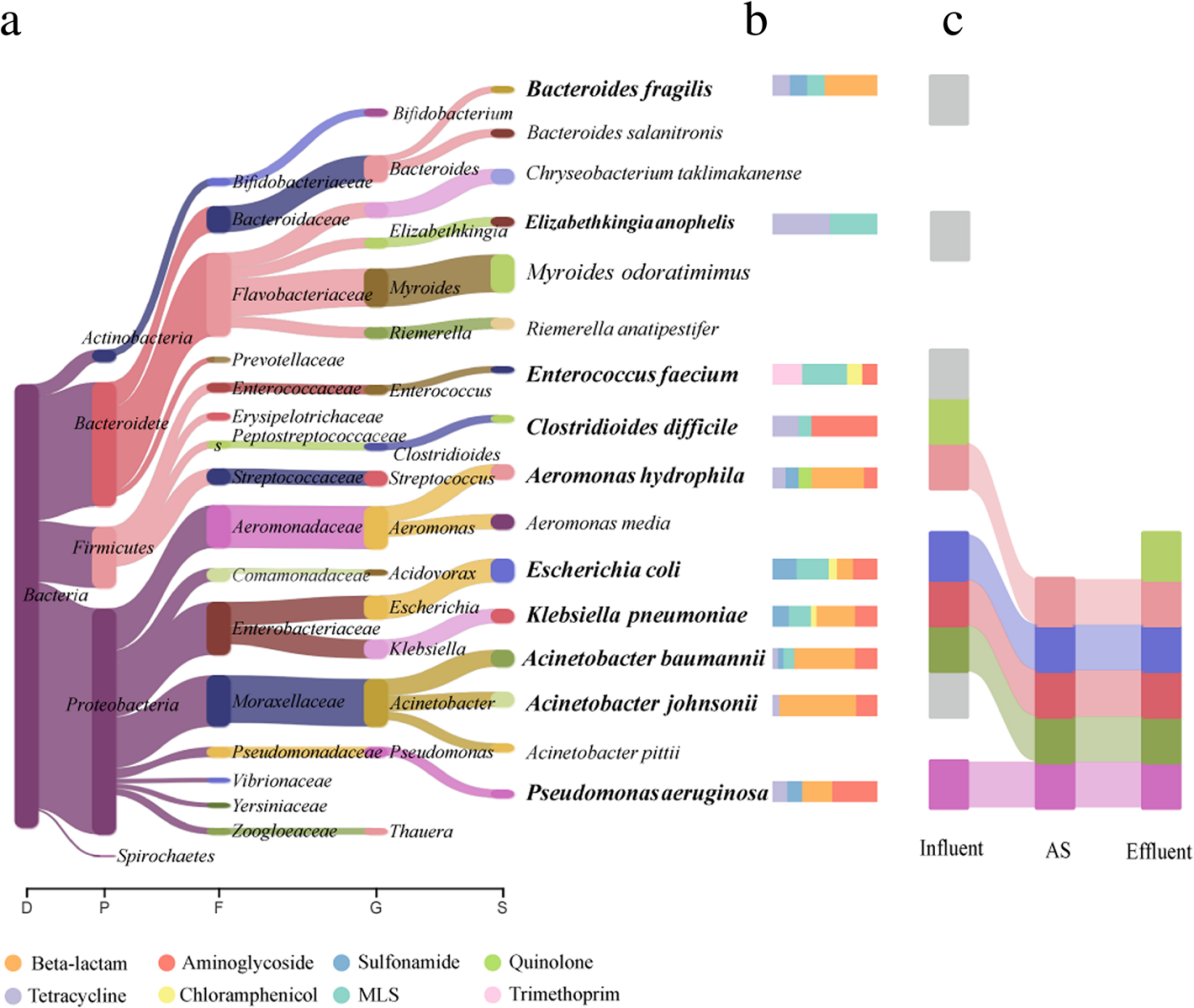
Host range of antibiotic resistance genes (ARGs) and the fate of antibiotic resistance pathogens (ARPs) across wastewater treatment compartments. **a** The phylogenetic tree shows the composition and relative abundance of the ARGs hosts (the most abundant 16 species were shown). **b** The bar chart indicates the diversity and relative abundance of the ARGs (eight types) carried by the corresponding pathogens (highlight in bold) on the left panel. **c** The flow chart indicates the fate of the ARPs which were highlighted in bold in Fig. 3a throughout the treatment process (Influent, activated sludge, and effluent). Gray: represents the bacteria which were only detected in the influent

Rapid antimicrobial-resistant pathogens (ARPs) identification is necessary for effective pathogen control in wastewater treatment. The real-time nature and long reads of Nanopore sequencing allow for rapid identification and simultaneously fate tracking of potential ARPs in WWTPs. As shown in Fig. 3a, a total of 16 species with relative abundance greater than 2% were detected, of which 10 species were potential pathogenic bacteria, accounting for 48.7% of all the identified ARBs. All the identified potential ARPs were primarily affiliated with the *Gammaproteobacteria* class (74.4%), including *Aeromonas*, *Escherichia*, *Klebsiella*, *Acinetobacter*, and *Pseudomonas* (Fig. 3a). Remarkably, four species in the ESKAPE panel of pathogens, *Enterococcus faecium* (2.6% of ARBs), *Klebsiella pneumoniae* (5.5% of ARBs), *Acinetobacter baumannii* (6.6% ARBs), and *Pseudomonas aeruginosa* (3.3% ARBs), harboring a high diversity of ARGs (at least four types), were identified across the WWTPs, and the most abundant ARGs carried by these potential pathogens were those of beta-lactam, aminoglycoside, and MLS, accounting for 78.8% of the total detected ARGs (Fig. 3b). The prevalence of these potential ARPs in WWTPs, including carbapenem-resistant *Acinetobacter baumannii* [52], multidrug-resistant *Enterococcus faecium* [53], and *CTX-M*-producing *Klebsiella pneumoniae* [54], has been reported with culture-based techniques, which were usually time-consuming; however, as the leading cause of nosocomial infections throughout the world, reducing the detection time for these ESKAPE pathogens is critical for risk management. Nanopore sequencing can reveal the ARPs profile in less than 24 h after receiving a sample, which significantly reduces the time required from sample collection to results delivery, as demonstrated in real-time surveillance of microorganisms in the field [55, 56].

The fate of these potential ARPs was further investigated. As expected, the influent samples possessed the highest ARP diversity; however, five species, including the three ESKAPE pathogens (*Klebsiella pneumoniae*, *Acinetobacter baumanni*, and *Pseudomonas aeruginosa*), were found at all treatment stages (Fig. 3c), whereas *Clostridium difficile* was only detected in influent and effluent. These results indicated that a variety of ARPs had high potential to pass the treatment process of WWTPs and enter the receiving environments and highlighted the importance of effective effluent disinfection, particularly considering their regrowth and reactivation, which have been confirmed by several studies [57, 58].

### Persistence of bacteria carrying plasmids encoding multidrug-resistance

**Fig. 4:**
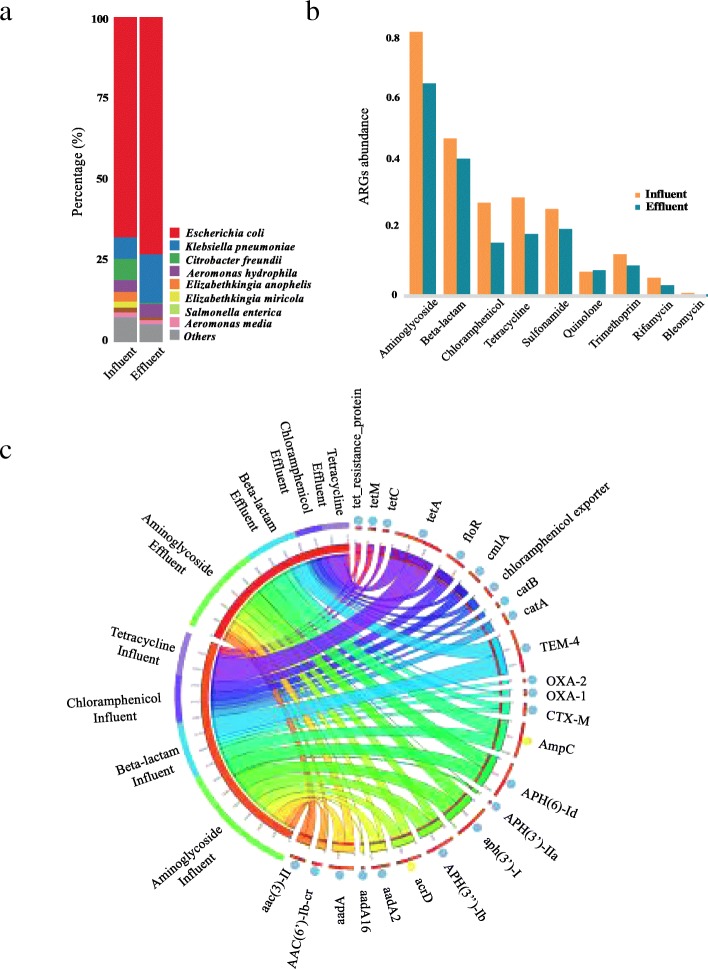
Comparison of phylogenetic taxonomic affiliation and ARGs profile between influent and effluent multidrug-resistant cultures. **a** The composition and relative abundance of multidrug-resistant bacteria. **b** The diversity and abundance of ARG types detected in the influent and effluent multidrug-resistant cultures, ARG number per million base pairs was used for ARGs quantification. **c** Distribution pattern and genetic location of four ARG types (aminoglycoside, beta-lactam, tetracycline, and chloramphenicol). The outmost dots are colored according to the genetic location of the corresponding ARG subtype (i.e., blue: carried by plasmids; yellow: located on the chromosome), the width of inner ribbon represents the relative abundance of ARG subtypes in the two samples

Phenotype in combination with genotype was used for further verification of the persistence of bacteria carrying plasmids encoding multidrug-resistance in the WWTPs. Bacteria that can simultaneously confer resistance to four different families of antibiotics, including ampicillin, kanamycin, tetracycline, and chloramphenicol, were identified from both influent and effluent cultures of the three WWTPs. As shown in Fig. 4a, the identified cultures were primarily affiliated with eight species based on the analysis of Nanopore sequencing data, including *Escherichia coli*, *Klebsiella pneumoniae*, *Citrobacter freundii*, *Aeromonas hydrophila*, *Elizabethkingia anophelis*, *Elizabethkingia miricola*, *Salmonella enterica*, and *Aeromonas media*, most of which were human pathogenic bacteria. The most dominant member was *Escherichia coli* in both influent and effluent cultures, accounting for 67.5% and 72.9% of all the identified resistant bacteria, respectively. Some bacteria in the influent were removed effectively during the treatment process, such as species of *Citrobacter* and *Elizabethkingia* (Fig. 4a). Additionally, four species, *Aeromonas hydrophila*, *Salmonella enterica*, *Klebsiella pneumoniae*, and *Aeromonas media*, were detected in both influent and effluent cultures, which indicated they were persistent across the treatment process. Moreover, the relative abundance of *Klebsiella pneumoniae* increased in the effluent cultures (from 6.7% to 15%). To verify the accuracy of microbial community analysis based on Nanopore sequencing, Illumina metagenomic sequencing was performed for the mixed influent multidrug-resistant cultures. As shown in Additional file 1: Figure S3a, the taxonomy result generated by Centrifuge [25] with Illumina sequencing data was largely in agreement at the dominant species level with that obtained by Nanopore sequencing. Meanwhile, consistency was observed regarding the taxonomic classification result at family level when using metagenomic sequences and 16S rRNA (V4 and V6 regions) genes (Additional file 1: Figure S3b). In addition, near-full-length 16S rRNA sequences were reconstructed successfully for those species with relative high abundance (Additional file 1: Table S5). Although the community complexity of the multidrug-resistant bacteria cultured from influent and effluent was greatly reduced due to the selective pressure from combined antibiotics, it is worth to point out the possible biases in profiling the microbial community caused by the shallow Nanopore sequencing library, especially for those with low abundance.

The resistance gene profiles were highly correlated with the resistance phenotypes to the given antibiotics, with aminoglycoside as the most abundant ARG, followed by beta-lactam, tetracycline, and chloramphenicol. Five types of ARGs associated with sulfonamide, quinolone, trimethoprim, rifamycin, and bleomycin, which were not used in the selective media, were also detected (Fig. 4b), highlighting the coexistence of ARGs in these resistant bacteria in the WWTPs.

As shown in Fig. 4b, more aminoglycoside and beta-lactam resistance genes were carried by these resistant bacteria than those resistant to tetracycline and chloramphenicol. Notably, a high abundance of sulfonamide resistance genes was observed in both influent and effluent cultures, suggesting their prevalence and persistence in multidrug-resistant bacteria in the WWTPs. Regarding the ARG subtypes, the resistance patterns showed a substantial similarity between the influent and effluent cultures (Fig. 4c); most importantly, except for two chromosomal encoding resistance genes (*acrD* and *AmpC*), all other ARGs encoding resistance to the four screening antibiotics were carried by plasmids, demonstrating that the persistent antibiotic resistance was primarily conferred by plasmids. Among the detected ARG types, more aminoglycoside subtypes were detected (ten subtypes) than those of beta-lactam (five subtypes), chloramphenicol (five subtypes), and tetracycline (four subtypes) (Fig. 4c). The most abundant subtypes in each ARG type, i.e., *aph*(*3*′)-*I*, *TEM*-*4*, *floR*, and *tetA*, were responsible for resistance to the four antibiotics we used. However, several aminoglycoside subtypes, such as *aph*(*3*″)-*Ib*, *aadA*, and *aph*(*6*)-*Id*, were also detected, but each of these is predicted to cause resistance to streptomycin instead of kanamycin, suggesting that aminoglycoside resistance genes were more likely to co-occur with other subtypes of ARGs, which was consistent with the high abundance observed in Fig. 4b.

It is necessary to point out that since it is not currently possible to build consensus Nanopore sequences to increase the accuracy, raw unpolished reads were used to generate AMR profiles for the mixed cultures, as well as for the environmental samples analyzed above. This may cause ARGs annotation biases to some extent. However, we believe this approach should be a useful start for efficient antibiotic resistome profiling and could be further improved.

**Fig. 5:**
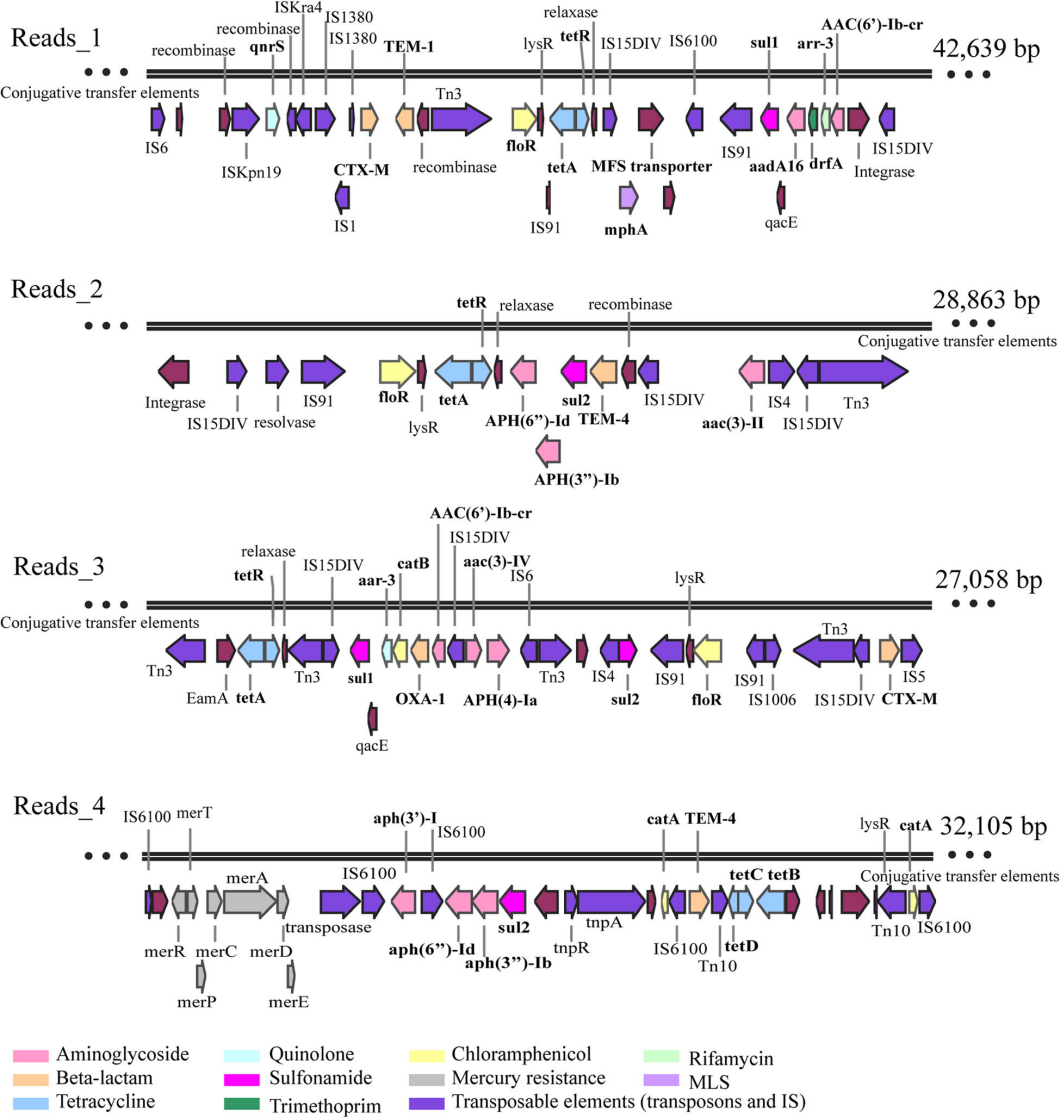
Schematic of the genetic organization of multidrug resistance gene cluster. Colors depict predicted ARGs categorization, as indicated

At last, the arrangement of ARGs located on plasmids was investigated. Given the antibiotics used in this study, only reads encoding at least four types of ARGs simultaneously in both influent and effluent cultures were investigated (Fig. 5). Genetic analysis of these reads showed genes involved in plasmid conjugation (i.e., relaxase and type IV secretion system), implying the prevalence of conjugative plasmids in the multidrug-resistant bacteria. Indeed, multiple ARGs are often co-localized on the same conjugative plasmids, which allows for the relatively easy spread of multidrug-resistance [59]. For example, reads_1 (42,639 bp) carried 11 ARGs, which could confer resistance to multiple antibiotic classes, including beta-lactam (*CTX*-*M* and *TEM*-*1*), aminoglycoside [*AAC*(*6*′)-*Ib*-*cr* and *aadA16*], chloramphenicol (*floR*), tetracycline (*tetA*), quinolone (*qnrS*), sulfonamide (*sul1*), rifampicin (*aar*-*3*), trimethoprim (*drfA*), and macrolides (*mphA*). Additionally, a complete class 1 integron carrying *AAC*(*6*′)-*Ib*-*cr*, *aar*-*3*, *drfA*, and *aadA16* was identified. Such complex structures of a multidrug-resistance gene cluster were easily resolved based on Nanopore ultra-long reads. Moreover, the co-occurrence of different aminoglycoside ARG subtypes was detected on the same plasmid (Fig. 5), such as the combination of three subtypes (*APH*(*6*)-*Id*, *APH*(*3*″)-*Ib*, and aph(3′)-I) on read_4 and similar arrangements on other reads. In addition to the co-occurrence pattern between ARG subtypes, Nanopore long sequences also identified the gene cluster encoding mercury resistance (*merA*, *merC*, *merD*, *merE*, *merP*, and *merR*) (shown on reads_4 in Fig. 5). Most strikingly, many types of insertion sequences (IS) and transposable (Tn) elements displayed a mosaic distribution on these conjugative plasmids, which may increase the probability of HGT. In fact, these abundant repetitive elements also make it difficult to assemble the Illumina reads into long contigs sufficient to elucidate the arrangement of complex ARG clusters [60]. This difficulty could be largely overcome by Nanopore sequencing technology as demonstrated in this study.

### Resistome quantification based on Nanopore and Illumina sequencing reads

The abundance of major ARG types (in terms of ARG number per million base pairs) revealed by Nanopore sequencing was compared to that revealed by Illumina sequencing. Overall, the quantitative results of the major ARGs calculated based on these two sequencing platforms were comparable as indicated by the Pearson correlation analyses (Additional file 1: Figure S2), except for two activated sludge samples (i.e., STAS and SWHAS). Discrepancies observed in activated sludge samples could be mainly originated from the limited sequencing depths, particularly considering the highly complex community with relatively low ARGs abundance in the activated sludge. (Additional file 1: Table S2). Such disagreements in ARGs quantification using different sequencing strategies have also been reported in previous studies where some ARG subtypes were only detected by Nanopore sequencing and some other ARG subtypes were only identified via Illumina sequencing [13]. Additionally, sequencing platform biases [61] and different ARG prediction algorithms adopted by these two sequencing platforms might also influence the ARGs quantification outputs, as Nanopore sequencing data analysis was based on tools designed for alignment of long high-error-rate sequences, whereas Illumina algorithm was based on BLAST similarity search.

## Conclusions

We reported the first workflow combining both Nanopore and Illumina sequencing technologies to comprehensively profile the genetic context of ARGs as well as to track their hosts across the wastewater treatment process. The results showed that MGEs (plasmids and ICEs)-associated ARGs dominated the resistome in WWTPs and their relative abundance increased in the effluent. Nanopore long reads greatly facilitated the characterization of multidrug-resistant conjugative plasmids. The significant role of these plasmids in facilitating the survival and persistence of multidrug-resistant bacteria in WWTPs was further confirmed by phenotype-genotype analysis. In summary, this work established a baseline framework for future studies related to mobile antibiotic resistome in the environment.

## Additional file

Additional file 1: **Table S1.** Summary statistics for reads generated by Nanopore (numbers in bold) and Illumina assemblies. Table S2. Summary of ARGs-carrying contigs after Illumina assembly and long reads generated by Nanopore sequencing. Table S3. Distribution and relative abundance of ARGs only detected by Illumina sequencing, “√” indicates the ARGs type detected in the corresponding samples. Table S4. Genetic location of major ARGs predicted from all Illumina assembled contigs. Table S5 Summary for the near-full-length 16S rRNA sequences reconstructed from mixed influent cultures using EMIRGE. Figure S1. Overview of the reads length (a), reads number (b) and average base call quality score (c) of the eleven Nanopore metagenomics datasets. Figure S2. Correlation analysis of major ARGs abundance (ARGs number per million base pairs) quantified based on Illumina sequencing and Nanopore reads, x-axis and y-axis represents the ARGs number calculated by Illumina and Nanopore datasets respectively. Figure S3. Comparison of phylogenetic taxonomic affiliation at species (a) and family level (b) between Illumina and Nanopore sequencing platforms for the mixed influent multidrug-resistant cultures. (DOCX 1263 kb)
