# Supplementary material for: Mobile antibiotic resistome in wastewater treatment plants revealed by Nanopore metagenomic sequencing
Source: Microbiome. 2019 Mar 21;7:44. doi: 10.1186/s40168-019-0663-0 (PMC6429696; doi:10.1186/s40168-019-0663-0)
Supplement: Supplementary file 1 — Table S1. Summary statistics for reads generated by Nanopore (numbers in bold) and Illumina assemblies. Table S2. Summary of ARGs-carrying contigs after Illumina assembly and long reads generated by Nanopore sequencing. Table S3. Distribution and relative abundance of ARGs only detected by Illumina sequencing, “√” indicates the ARGs type detected in the corresponding samples. Table S4. Genetic location of major ARGs predicted from all Illumina assembled contigs. Table S5 Summary for the near-full-length 16S rRNA sequences reconstructed from mixed influent cultures using EMIRGE. Figure S1. Overview of the reads length (a), reads number (b) and average base call quality score (c) of the eleven Nanopore metagenomics datasets. Figure S2. Correlation analysis of major ARGs abundance (ARGs number per million base pairs) quantified based on Illumina sequencing and Nanopore reads, x-axis and y-axis represents the ARGs number calculated by Illumina and Nanopore datasets respectively. Figure S3. Comparison of phylogenetic taxonomic affiliation at species (a) and family level (b) between Illumina and Nanopore sequencing platforms for the mixed influent multidrug-resistant cultures. (DOCX 1263 kb) [file 40168_2019_663_MOESM1_ESM.docx]

**Supporting information**

**Mobile antibiotic resistome in wastewater treatment plants revealed by Nanopore metagenomic sequencing**

You Che, Yu Xia, Lei Liu, An-Dong Li, Yu Yang, Tong Zhang*

Environmental Biotechnology Laboratory, The University of Hong Kong, Hong Kong

| Sample | Long reads/Contigs number | Meanlen (bp) | Maxlen(bp) | N50 (bp) | Data size (G) |
| --- | --- | --- | --- | --- | --- |
| STIN | **627701**\|552561 | **3921**\|1391 | **31490**\|191521 | **5872**\|1642 | **2.5**\|18.0 |
| STAS | **527395**\|568206 | **5059**\|1456 | **21559**\|983527 | **7029**\|1734 | **2.7**\|16.0 |
| STEFF | **720148**\|349071 | **4667**\|1478 | **54416**\|328115 | **7340**\|1793 | **3.4**\|13.37 |
| SWHIN | **586169**\|456350 | **4846**\|1352 | **33567**\|307628 | **7833**\|1539 | **2.8**\|16.73 |
| SWHAS | **522782**\|518038 | **5656**\|1354 | **45452**\|220064 | **9044**\|1549 | **3.0**\|12.38 |
| SWHEFF | **839725**\|408067 | **5054**\|1462 | **52163**\|957514 | **7899**\|1777 | **4.2**\|13.73 |
| STLIN | **469061**\|334433 | **6302**\|1267 | **73530**\|1130099 | **10183**\|1383 | **3.0**\|11.3 |
| STLAS | **739597**\|515946 | **7329**\|1498 | **48719**\|1068336 | **10674**\|1863 | **5.4**\|15.2 |
| STLEFF | **703599**\|359463 | **4620**\|1383 | **46566**\|1416200 | **7167**\|1538 | **3.3**\|13.7 |
| IN_multi_resistance | **378859** | **11841** | **109176** | **18123** | **4.5** |
| EFF_multi_resistance | **326972** | **10200** | **95103** | **15034** | **3.3** |

**Additional file 1: Table S1** Summary statistics for reads generated by Nanopore (numbers in bold) and Illumina assemblies. Nine samples (influent, activated sludge and effluent from three WWTPs) were sequenced by Nanopore and assembled using Illumina reads whereas no assembly was performed for multidrug-resistant bacteria cultured from influent and effluent.

Abbreviation: STIN, Shatin STP influent; STAS, Shatin STP activated sludge; STEFF, Shatin STP effluent; SWHIN, Shek Wu Hui STP influent, SWHAS, Shek Wu Hui STP activated sludge; SWHEFF, Shek Wu Hui STP effluent; STLIN, Stanley STP influent; STLAS, Stanley STP activated sludge; STLEFF, Stanley STP effluent.

**Table S2**. Summary of ARGs-carrying contigs after Illumina assembly and long reads generated by Nanopore sequencing. Green: Illumina assembly result; yellow: Nanopore sequencing result.

| Sample | Contigs number  (after assembly) | ARGs-carrying contigs number | Average ARGs-carrying contigs length (bp) | Nanopore reads number | ARGs-carrying reads number | Average ARGs-carrying reads length (bp) |
| --- | --- | --- | --- | --- | --- | --- |
| STIN | 552561 | 75 | 2944 | 627701 | 255 | 6443 |
| STAS | 568206 | 13 | 2398 | 527395 | 87 | 6586 |
| STEFF | 349071 | 22 | 2900 | 720148 | 114 | 7211 |
| SWHIN | 456350 | 83 | 3748 | 586169 | 478 | 7643 |
| SWHAS | 518038 | 7 | 2626 | 522782 | 36 | 9461 |
| SWHEFF | 408067 | 13 | 3246 | 839725 | 106 | 7915 |
| STLIN | 334433 | 58 | 2944 | 469061 | 509 | 10372 |
| STLAS | 515946 | 12 | 1662 | 739597 | 68 | 8966 |
| STLEFF | 359463 | 33 | 3433 | 703599 | 138 | 11734 |

**Table S3**. Distribution and relative abundance of ARGs only detected by Illumina sequencing, “√” indicates the ARGs type detected in the corresponding samples.

| **ARGs type** | **STIN** | **STAS** | **STEFF** | **SWHIN** | **SWHAS** | **SWHEFF** | **STLIN** | **STLAS** | **STLEFF** |
| --- | --- | --- | --- | --- | --- | --- | --- | --- | --- |
| bacitracin |  | √ | √ | √ | √ | √ |  |  |  |
| fosfomycin | √ |  | √ | √ |  |  |  |  |  |
| fosmidomycin | √ | √ | √ |  | √ | √ |  | √ | √ |
| kasugamycin | √ |  | √ | √ |  | √ | √ |  | √ |
| multidrug |  |  |  |  |  |  |  | √ |  |
| polymyxin | √ |  | √ | √ | √ | √ |  |  | √ |
| puromycin | √ | √ |  |  |  |  |  |  |  |
| quinolone |  |  |  |  | √ | √ |  |  |  |
| rifamycin | √ |  |  |  |  |  |  |  | √ |
| tetracenomycin_C | √ |  |  | √ |  | √ |  |  |  |
| trimethoprim |  |  |  |  | √ |  |  |  |  |
| vancomycin | √ |  | √ | √ | √ | √ | √ | √ | √ |
| Relative abundance (%) | **0.8** | **3.0** | **1.2** | **1.2** | **7.4** | **8.7** | **0.1** | **12** | **0.8** |

**Table S4**. Genetic location of major ARGs predicted from all Illumina assembled contigs.

| **ARGs type** | **Total ARGs number** | **Plasmids carrying** | **Chromosome carrying** | **Unclassified** |
| --- | --- | --- | --- | --- |
| aminoglycoside | 46 | 28 | 4 | 14 |
| beta-lactam | 78 | 45 | 10 | 23 |
| chloramphenicol | 22 | 13 | 1 | 8 |
| tetracycline | 44 | 16 | 15 | 13 |
| sulfonamide | 11 | 9 | 1 | 1 |
| MLS | 55 | 28 | 10 | 17 |
| quinolone | 6 | 3 | 0 | 3 |
| trimethoprim | 7 | 4 | 0 | 3 |
| multidrug | 32 | 0 | 24 | 8 |

| 16S rRNA sequences | Length (bp) | Similarity  ( best BLAST hit)  (%) | Relative abundance (%) | Species | Family |
| --- | --- | --- | --- | --- | --- |
| 1 | 1530 | 99.77 | 29.0 | *Escherichia coli* | *Enterobacteriaceae* |
| 2 | 1121 | 100 | 34.9 | *Escherichia coli* | *Enterobacteriaceae* |
| 3 | 1007 | 98.98 | 11.0 | *Citrobacter freundii* | *Enterobacteriaceae* |
| 4 | 1442 | 100 | 8.9 | *Aeromonas hydrophila* | *Aeromonadaceae* |
| 5 | 1482 | 99.60 | 2.5 | *Elizabethkingia anophelis* | *Flavobacteriaceae* |
| 6 | 1054 | 100 | 1.0 | *Chryseobacterium sp.* | *Flavobacteriaceae* |

**Table S5**. Summary for the near-full-length 16S rRNA sequences reconstructed from mixed influent cultures using EMIRGE. Relative abundance estimates for each assembled 16S rRNA gene were derived from the probabilistic accounting of reads in EMIRGE.


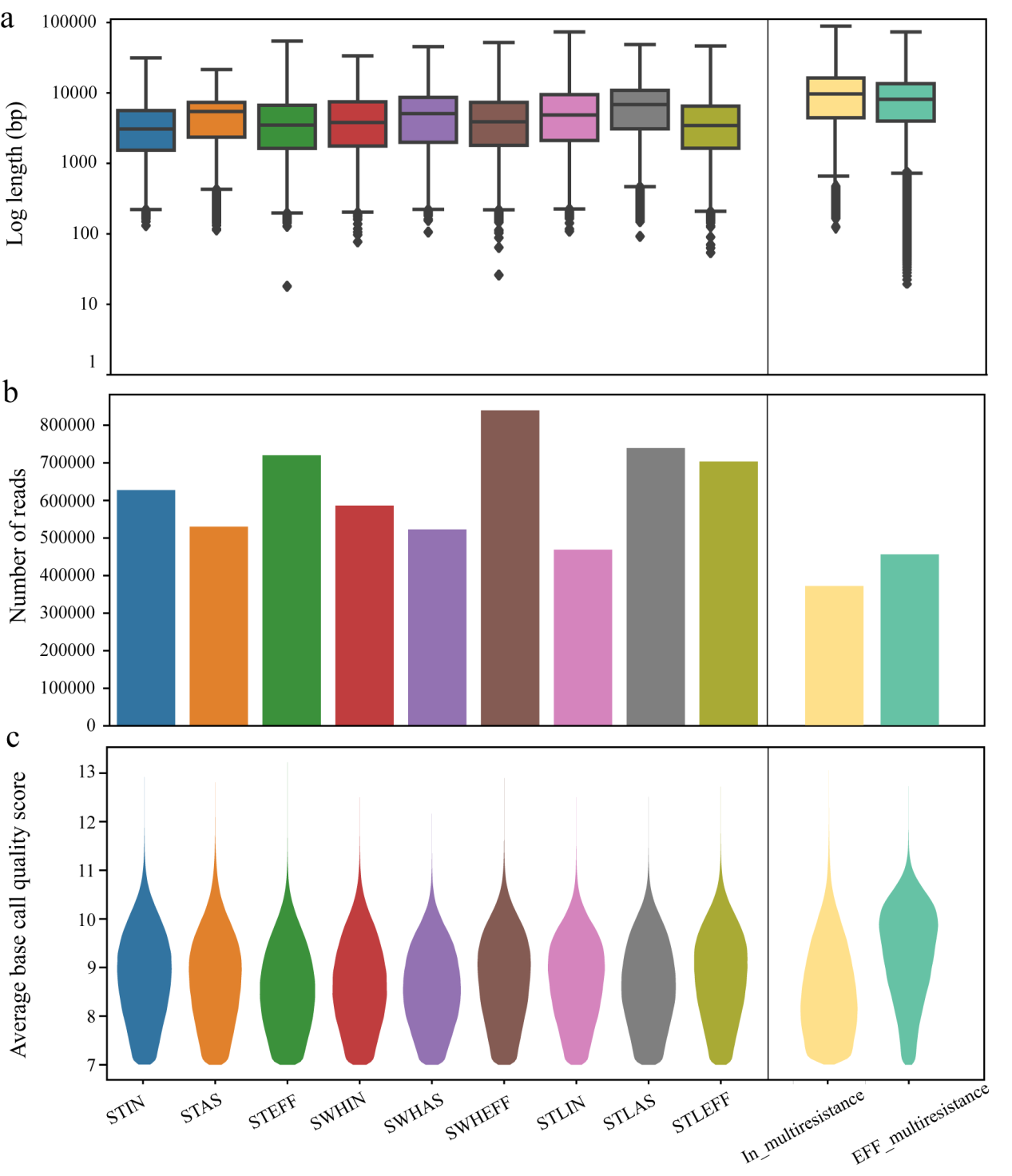


**Figure S1**. Overview of the reads length (a), reads number (b) and average base call quality score (c) of the eleven Nanopore metagenomics datasets. STIN, Shatin STP influent; STAS, Shatin STP activated sludge; STEFF, Shatin STP effluent; SWHIN, Shek Wu Hui STP influent, SWHAS, Shek Wu Hui STP activated sludge; SWHEFF, Shek Wu Hui STP effluent; STLIN, Stanley STP influent; STLAS, Stanley STP activated sludge; STLEFF, Stanley STP effluent; In_multiresistance, mix multidrug-resistant culture from three influent samples; EFF_multiresistance, mix multidrug-resistant culture from three effluent samples.

**Figure S2**. Correlation analysis of major ARGs abundance (ARGs number per million base pairs) quantified based on Illumina sequencing and Nanopore reads, x-axis and y-axis represents the ARGs number calculated by Illumina and Nanopore datasets respectively.

**Figure S3**. Comparison of phylogenetic taxonomic affiliation at species (a) and family level (b) between Illumina and Nanopore sequencing platforms for the mixed influent multidrug-resistant cultures.
